# Designing rice panicle architecture via developmental regulatory genes

**DOI:** 10.1270/jsbbs.22075

**Published:** 2023-03-21

**Authors:** Ayumi Agata, Motoyuki Ashikari, Yutaka Sato, Hidemi Kitano, Tokunori Hobo

**Affiliations:** 1 Graduate School of Bioagricultural Sciences, Nagoya University, Nagoya, Aichi 464-8601, Japan; 2 National Institute of Genetics, Mishima, Shizuoka 411-8540, Japan; 3 Bioscience and Biotechnology Center, Nagoya University, Nagoya, Aichi 464-8601, Japan

**Keywords:** rice, panicle development, panicle architecture, near-isogenic lines, grain filling

## Abstract

Rice panicle architecture displays remarkable diversity in branch number, branch length, and grain arrangement; however, much remains unknown about how such diversity in patterns is generated. Although several genes related to panicle branch number and panicle length have been identified, how panicle branch number and panicle length are coordinately regulated is unclear. Here, we show that panicle length and panicle branch number are independently regulated by the genes *Prl5*/*OsGA20ox4*, *Pbl6*/*APO1*, and *Gn1a*/*OsCKX2*. We produced near-isogenic lines (NILs) in the Koshihikari genetic background harboring the elite alleles for *Prl5*, regulating panicle rachis length; *Pbl6*, regulating primary branch length; and *Gn1a*, regulating panicle branching in various combinations. A pyramiding line carrying *Prl5*, *Pbl6*, and *Gn1a* showed increased panicle length and branching without any trade-off relationship between branch length or number. We successfully produced various arrangement patterns of grains by changing the combination of alleles at these three loci. Improvement of panicle architecture raised yield without associated negative effects on yield-related traits except for panicle number. Three-dimensional (3D) analyses by X-ray computed tomography (CT) of panicles revealed that differences in panicle architecture affect grain filling. Importantly, we determined that *Prl5* improves grain filling without affecting grain number.

## Introduction

The rice (*Oryza sativa* L.) panicle is a branched structure that produces grains (Supplemental Fig. 1). The generated branching patterns directly reflect rice grain productivity. Indeed, slight changes in the number and/or length of branches result in a diversity of panicle morphological patterns in natural rice variations. Understanding how such diverse architectures arise will reveal the underlying developmental process of the rice panicle. Furthermore, achieving an ideal panicle architecture using developmental regulatory genes could enhance rice yield potential (Sreenivasulu *et al.* 2021).

Increasing evidence has revealed that numerous factors, such as transcription factors and plant hormones, play critical roles in determining panicle morphology in rice (Deveshwar *et al.* 2020, Kellogg 2022, Zhang and Yuan 2014). Numerous findings have been accumulated, particularly through quantitative trait locus (QTL) analysis exploiting the diversity of natural rice variations. Several genes have been cloned that affect branching number (Ashikari *et al.* 2005, Fujishiro *et al.* 2018, Ikeda *et al.* 2005, 2007, Komatsu *et al.* 2003, Miura *et al.* 2010, Ookawa *et al.* 2010, Wu *et al.* 2016). For example, *Grain number 1a* (*Gn1a*) encodes the cytokinin oxidase/dehydrogenase OsCKX2 (Ashikari *et al.* 2005). Lower *Gn1a* expression results in more branches due to the greater accumulation of cytokinins in the panicle. The mechanism that controls panicle size had remained unexplored for a long time, but we recently clarified some of the salient details (Agata *et al.* 2020). *PANICLE RACHIS LENGTH5* (*Prl5*) encodes a gibberellin biosynthesis enzyme, GA 20-oxidase 4 (OsGA20ox4). Higher *Prl5* expression is associated with the elongation of the panicle rachis. *PRIMARY BRANCH LENGTH6* (*Pbl6*) is allelic to *ABERRANT PANICLE ORGANIZATION1* (*APO1*), which encodes an F-box protein. We demonstrated that higher expression of *Pbl6* is responsible for primary branch elongation. *Pbl6* also pleiotropically affects panicle branching and panicle length (Agata *et al.* 2020, Ookawa *et al.* 2010). We have previously shown how panicle length can be freely designed by independently controlling the length of the panicle rachis and the primary branch. Isolated findings about the genetic mechanisms related to the number or length of panicle branches have accumulated. However, fundamental questions remain: Are the panicle branches and panicle length coordinately regulated, and if so, how? Elucidating the mechanisms of this coordinated regulation would provide greater understanding of panicle development and offer guidance for plant breeding practices.

During the past decade, numerous agronomically important QTLs have been identified, of which *Gn1a*, *Prl5*, and *Pbl6* are examples (Ikeda *et al.* 2010, Li *et al.* 2018). Because allele pyramiding is a useful strategy for generating new varieties by combining distinct alleles corresponding to individual QTLs into one line (Ashikari and Matsuoka 2006, Hasan *et al.* 2015, Takeda and Matsuoka 2008), many attempts have been made to produce crop plants with high productivity by combining such QTLs (Septiningsih *et al.* 2009, Singh *et al.* 2001, Suh *et al.* 2015, Tao *et al.* 2016, Zeng *et al.* 2017). However, many allele combinations exhibited a trade-off between grain yield and other key yield components. For example, *Ideal Plant Architecture 1* (*IPA1*, also identified as *Wealthy Farmer’s Panicle* [*WFP*]) plays opposite roles in determining tiller number and panicle branch number (Jiao *et al.* 2010, Miura *et al.* 2010). *TEOSINTE BRANCHED1* (*TB1*) pleiotropically regulates spikelet number and tiller number, resulting in a trade-off between these traits (Yano *et al.* 2015). *DENSE AND ERECT PANICLE1* (*DEP1*) encodes a phosphatidylethanolamine-binding protein (PEBP)-like protein. A gain-of-function mutation, *dep1*, results in decreased inflorescence internode length and increased spikelet number to produce a dense and erect panicle (Huang *et al.* 2009), indicating that panicle branching and panicle length can also be subjected to trade-offs.

In this study, we produced near-isogenic lines (NILs) with different panicle architecture by introducing *Prl5* and *Pbl6*, which control panicle length, and *Gn1a*, which regulates the number of branches. We evaluated the effects of the three genes on panicle branching patterns and other yield traits. Furthermore, we visualized the changes in panicle branching patterns on grain filling by three-dimensional (3D) analyses using X-ray computed tomography (CT) of panicles. We examined the relationship between regulatory mechanisms of panicle branching number and panicle size and discuss the possibility of using developmental regulatory genes in rice breeding.

## Materials and Methods

### Plant materials

The rice (*Oryza sativa* L) cultivar ‘ST-1’ was selected from the rice collection of the Field Science Center at Nagoya University. An ST-1 × Koshihikari F_1_ plant was backcrossed six times to ‘Koshihikari’ to generate near-isogenic lines. The lines NIL-*Prl5*^ST-1^, NIL-*Pbl6*^ST-1^, NIL-*Gn1a*^ST-1^, NIL-*Prl5*^ST-1^ + *Pbl6*^ST-1^, NIL-*Prl5*^ST-1^ + *Gn1a*^ST-1^, NIL-*Pbl6*^ST-1^ + *Gn1a*^ST-1^, and NIL-*Prl5*^ST-1^ + *Pbl6*^ST-1^ + *Gn1a*^ST-1^ were selected from the BC_6_F_2_ population using simple sequence repeat (SSR) markers (Supplemental Table 1). All materials were grown under natural conditions in a paddy field at the Field Science Center at Nagoya University, Togo, Aichi, Japan, in 2020. Seeds were germinated in a seedbed in early May and transplanted to the field using one seedling per hill in late June. The planting density was with a spacing of 25 × 25 cm. Fertilizer was applied at about 8 kg N per 10 are.

### Observation of panicle architecture

The main panicle was measured from each plant for observation. Panicle length, panicle rachis length, primary branch length, number of primary branches, secondary branches, and total grain number were visually measured.

### Evaluation of yield-related traits

After 30 days of the heading date, the whole plant was sampled and air-dried for about two weeks before evaluation. Culm length, panicle number per plant, main panicle weight, panicle weight per plant, and thousand-grain weight were measured. The measured grains were randomly selected from the whole panicle. Well-filled and hulled grains were dried down to under a moisture content of 14% and measured.

### 3D analysis by X-ray CT

Main panicles were harvested from plants 30 days after the heading date and were dried down to under a moisture content of 14%. Main panicles were scanned using a micro-focus X-ray CT (ScanXmate-L090T, Comscan, Japan) at a tube voltage peak of 40 kV and a tube current of 200 μA. Samples were rotated 360° in steps of 0.3°, generating 1,200 projection images of 1,296 × 1,152 pixels. The CT data were reconstructed at an isotropic resolution of 33.1 × 33.1 × 33.1 μm^3^. Each grain volume was analyzed based on 3D volume-rendering data using Fiji software (3D object counter) (Schindelin *et al.* 2012).

### Statistical analysis and reproducibility

All experiments were conducted in at least three biological replicates to ensure reproducibility. The sample numbers are indicated in the figure legends. Means from at least three independent biological replicates are presented in each figure, with error bars representing standard deviation. The number (*n*) indicates the number of biological replicates in the figure legends. Statistical analysis was performed in R software (https://www.r-project.org/). Statistical differences were determined by one-way analysis of variance with multi-comparison Tukey’s HSD post-hoc test. A *P* value <0.05 was considered to be statistically significant. Significant differences are indicated by asterisks in the figures.

### Data and materials availability

All data are available in the main text or supplemental materials.

## Results

### Development of near-isogenic lines with various panicle branching patterns

**Fig. 1.**
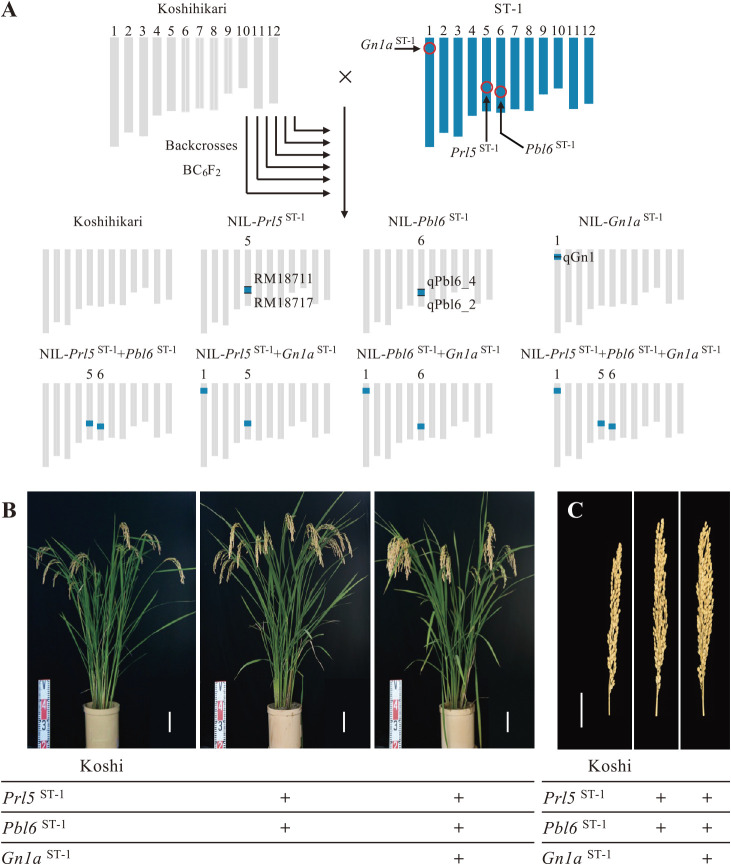
Graphical genotypes of near-isogenic lines. (A) Schematic diagrams of the parental lines Koshihikari (chromosomes shown in gray), ST-1 (chromosomes shown in blue), and their derived near-isogenic lines (NILs). Red circles indicate the genomic coordinates of *Gn1a*, *Prl5*, and *Pbl6*. (B) Gross morphologies of Koshihikari, NIL-*Prl5*^ST-1^ + *Pbl6*^ST-1^, and NIL-*Gn1a*^ST-1^ + *Prl5*^ST-1^ + *Pbl6*^ST-1^. Scale bar, 10 cm. (C) Representative panicles of Koshihikari, NIL-*Prl5*^ST-1^ + *Pbl6*^ST-1^, and NIL-*Gn1a*^ST-1^ + *Prl5*^ST-1^ + *Pbl6*^ST-1^. Scale bar, 5 cm.

To gain more insight into the precise relationship between panicle branching and panicle length, we produced pyramided NILs carrying the *Gn1a*, *Prl5*, and/or *Pbl6* genomic regions from ST-1 in an otherwise Koshihikari background by crossing ST-1 to Koshihikari, followed by repeated backcrosses to Koshihikari. The ST-1 alleles of *Prl5* and *Pbl6* are more highly expressed than their Koshihikari counterparts, resulting in a longer panicle rachis length (*Prl5*) and in a longer primary branch length (*Pbl6*). We sequenced the *Gn1a* region from ST-1 and identified the same mutation harbored by a known allele from the rice *indica* variety ‘Habataki’ (Ashikari *et al.* 2005). We selected seven sets of NILs from the BC_6_F_2_ population based on their genotypes at *Prl5*, *Pbl6*, and *Gn1a* using simple sequence repeat (SSR) markers (Fig. 1A). A 58 kb ST-1 region containing *Prl5* was introduced into Koshihikari using RM18711 and RM18717. A 6 kb ST-1 region containing *Pbl6* was introduced using qPbl6_4 and qPbl6_2. *Gn1a* region of ST-1 was introduced using qGn1. Overall morphology did not differ between Koshihikari and the NILs (Fig. 1B), but panicle morphologies appeared different among them (Fig. 1C).

### Panicle length and branching are independently controlled

**Fig. 2.**
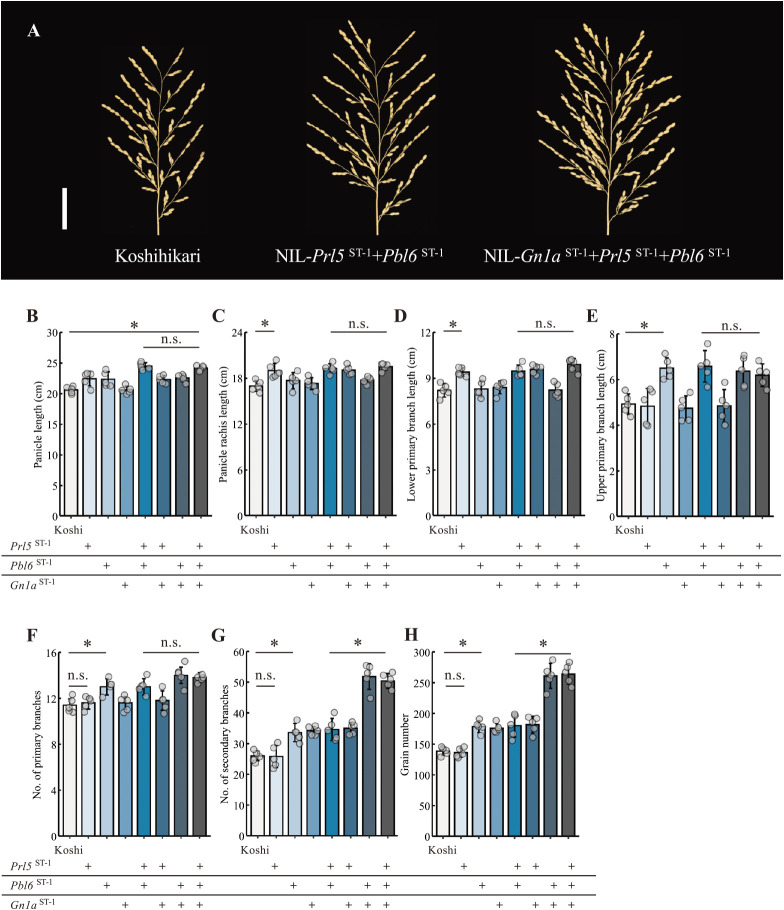
Effects of QTL pyramiding on each panicle trait. (A) Representative panicle morphology of Koshihikari, NIL-*Prl5*^ST-1^ + *Pbl6*^ST-1^, and NIL-*Gn1a*^ST-1^ + *Prl5*^ST-1^ + *Pbl6*^ST-1^. Scale bar, 5 cm. (B–H) Panicle traits in Koshihikari and derived NILs. (B) Panicle length. (C) Panicle rachis length. (D) Average length of the lower three primary branches. (E) Average length of the upper three primary branches. (F) Number of primary branches. (G) Number of secondary branches. (H) Grain number. Data are shown as means ± SD (*n* = 5 plants). *, *P* < 0.05 by Tukey’s significant difference test.

NIL-*Prl5*^ST-1^ + *Pbl6*^ST-1^ showed a longer panicle, while NIL-*Gn1a*^ST-1^ + *Prl5*^ST-1^ + *Pbl6*^ST-1^ had a longer and more branched panicle than Ksohihikari (Fig. 2A). To evaluate the effects of all gene combinations on panicle architecture, we characterized each panicle organ in each NIL in detail. NIL-*Prl5*^ST-1^ + *Pbl6*^ST-1^ exhibited a longer panicle length than Koshihikari, as previously reported (Fig. 2B) (Agata *et al.* 2020). *Prl5*^ST-1^ affected panicle rachis length and lower primary branch length (Fig. 2C, 2D), and *Pbl6*^ST-1^ modified upper primary branch length (Fig. 2E). By contrast, *Gn1a*^ST-1^ displayed no effect on rachis or branch length (Fig. 2B–2E). *Prl5*^ST-1^ similarly did not change branch number, whereas *Pbl6*^ST-1^ pleiotropically affected primary branch length and branch number, resulting in increased grain number (Fig. 2F–2H). *Gn1a*^ST-1^ increased the number of secondary branches rather than the number of primary branches (Fig. 2F, 2G), thereby contributing to an increase in total grain number in the relevant NILs (Fig. 2H). Finally, NIL-*Gn1a*^ST-1^ + *Prl5*^ST-1^ + *Pbl6*^ST-1^ produced about 264 grains on average, which was about 1.9 times more than Koshihikari (Fig. 2H). Observations of the panicle traits in the NILs revealed that multiple panicle developmental genes independently regulate branch number and branch length without showing any trade-off relationship between number and length.

### Various combinations of alleles at *Prl5*, *Pbl6*, and *Gn1a* result in diverse panicle branching patterns

**Fig. 3.**
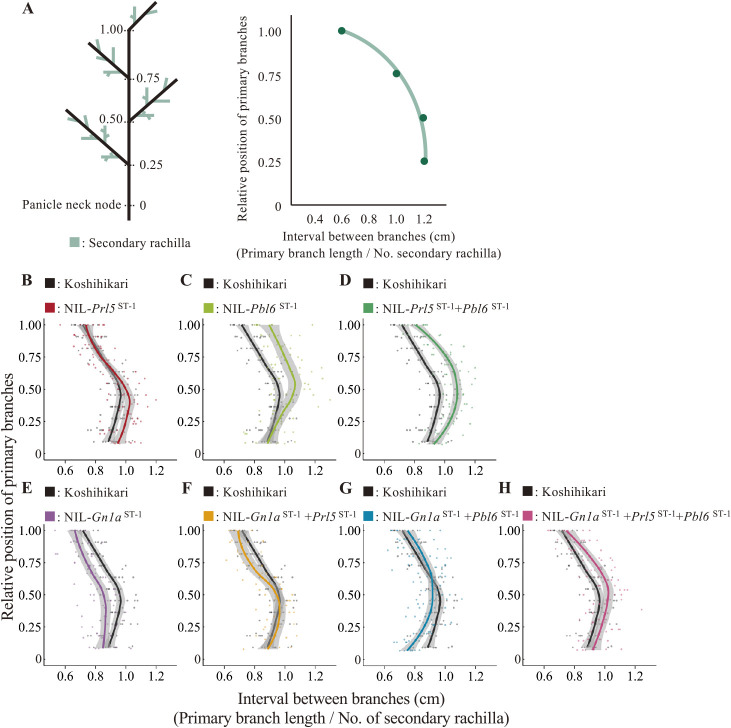
Effects of QTL pyramiding on panicle branching patterns. (A) Comparison of the interval between branches on each primary branch. The *y*-axis shows the relative position of each primary branch; the primary branch at the tip is scored as position 1. The value on the *x*-axis is the interval between branches on each primary branch. The values were calculated by dividing primary branch length by the number of secondary rachilla. Solid lines show the regression curve. (B–H) Interval between branches on each primary branch for Koshihikari and derived NILs. The black line and dots indicate Koshihikari; the color line and dots indicate the NIL: NIL-*Prl5*^ST-1^ (B), NIL-*Pbl6*^ST-1^ (C), NIL-*Prl5*^ST-1^ + *Pbl6*^ST-1^ (D), NIL-*Gn1a*^ST-1^ (E), NIL-*Prl5*^ST-1^ + *Gn1a*^ST-1^ (F), NIL-*Gn1a*^ST-1^ + *Pbl6*^ST-1^ (G), and NIL-*Gn1a*^ST-1^ + *Prl5*^ST-1^ + *Pbl6*^ST-1^ (H). All data in B–H are from *n* = 5–9 plants.

To assess whether the arrangement of grains over the whole panicle might take on different patterns by independently controlling the number and length of branching, we next examined panicle branching patterns in Koshihikari and the NILs. To this end, we calculated the length interval between secondary rachilla as an index of grain density by dividing the value of each primary branch length by the secondary rachilla number (Fig. 3A). When compared to the profile obtained for Koshihikari, we observed that *Prl5*^ST-1^ and *Pbl6*^ST-1^ elongate the basal and apical primary branch lengths, thus elongating the length interval between branches along the basal and apical sides, respectively (Fig. 3B, 3C). Combining these two alleles in the same background resulted in an additive elongation profile of the length interval between branches from the base to the tip (Fig. 3D). The *Gn1a*^ST-1^ allele, which does not affect length but increases branch number, shortened the length interval between branches from the base to the tip (Fig. 3E). When *Prl5*^ST-1^ or *Pbl6*^ST-1^ was combined with *Gn1a*^ST-1^, the length interval between branches elongated to the same extent as that seen in Koshihikari, due to the effect on primary branch elongation. We observed the effect of primary branch elongation at the base (*Gn1a*^ST-1^ + *Prl5*^ST-1^) or the tip (*Gn1a*^ST-1^ + *Pbl6*^ST-1^) of the panicle (Fig. 3F, 3G). Finally, by combining all three alleles, we determined that *Gn1a*^ST-1^ increases branch number, while *Prl5*^ST-1^ and *Pbl6*^ST-1^ extended primary branch length, resulting in the elongation of the length interval between branches from the base to the tip (Fig. 3H). Various combinations of *Prl5*, *Pbl6*, and *Gn1a* alleles showed different patterns of panicle architecture. This result suggests that *Prl5*, *Pbl6*, and *Gn1a* are key genes to generate a diversity of panicle morphologies in rice.

### Modifying panicle architecture enhances rice productivity

**Fig. 4.**
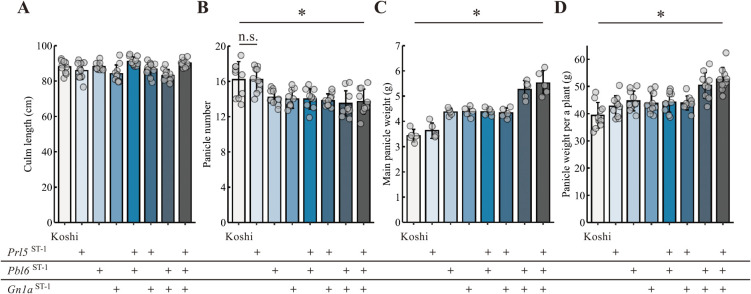
Effects of QTL pyramiding on yield-related traits. (A–D) Yield-related traits in Koshihikari and derived NILs: (A) culm length, (B) panicle number per plant, (C) main panicle weight, and (D) panicle weight per plant. Data are shown as means ± SD (*n* = 10 plants in A, B, and D, *n* = 5 plants in C). *, *P* < 0.05 by Tukey’s significant difference test.

To investigate whether a trade-off exists between grain productivity and other key yield components, we measured various yield-related traits. None of the three alleles at *Prl5*, *Pbl6*, or *Gn1a* affected culm length (Fig. 4A). However, with the exception of NIL-*Prl5*^ST-1^, panicle number decreased slightly with the introduction of more alleles (Fig. 4B). The main panicle weight of NIL-*Gn1a*^ST-1^ + *Prl5*^ST-1^ + *Pbl6*^ST-1^ was 5.52 g, which was about 1.6 times more than that of Koshihikari (Fig. 3C). Panicle weight per plant for NIL-*Gn1a*^ST-1^ + *Prl5*^ST-1^ + *Pbl6*^ST-1^ was 52.6 g, which was about 1.3 times more than that of Koshihikari (Fig. 3D). The increase of panicle weight per plant was modest compared to that measured for main panicle weight, likely because panicle number dropped slightly between the two genotypes (Fig. 3B–3D). Importantly, improved panicle architecture by combining three genes resulted in higher yield. This result suggests that developmental regulatory genes are useful for designing panicle architecture to enhance rice productivity.

### Panicle architecture influences grain filling

**Fig. 5.**
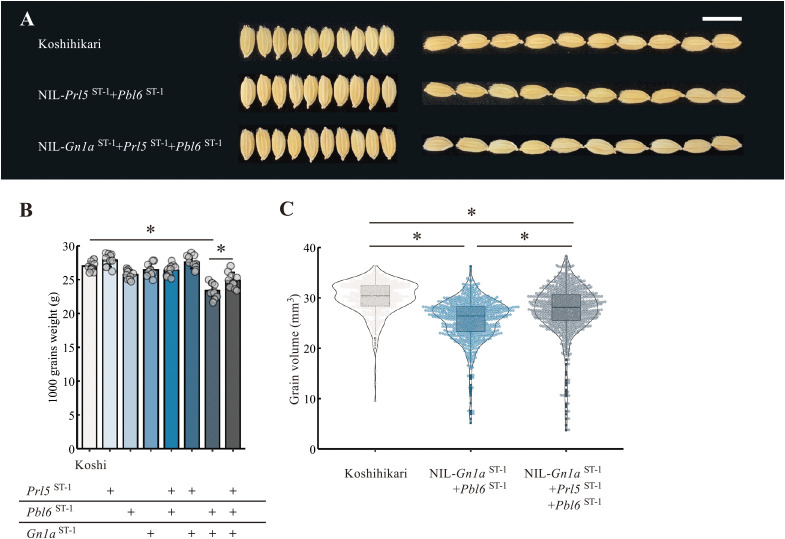
Effects of QTL pyramiding on grain traits. (A) Representative grains of Koshihikari and derived NILs NIL-*Prl5*^ST-1^ + *Pbl6*^ST-1^ and NIL-*Gn1a*^ST-1^ + *Prl5*^ST-1^ + *Pbl6*^ST-1^. Scale bars, 1 cm. (B) Thousand-grain weight (g) in Koshihikari and derived NILs. Data are shown as means ± SD (*n* = 10 plants). (C) Grain volumes, as analyzed by 3D X-ray CT, in Koshihikari, NIL-*Gn1a*^ST-1^ + *Pbl6*^ST-1^, and NIL-*Gn1a*^ST-1^ + *Prl5*^ST-1^ + *Pbl6*^ST-1^. *n* = 3 plants. The volumes of all grains for all three individuals per strain are plotted. *, *P* < 0.05 by Tukey’s significant difference test.

Although spikelet shape did not differ between Koshihikari and any of the NILs (Fig. 5A), the increase in main panicle weight was moderate compared to that of grain number (Figs. 2H, 4C), raising the possibility that grain filling decreases slightly following an increase in grain number. However, the precise effect of different panicle branching patterns on grain filling has not been clarified so far because of a lack of research materials. To explore the possible effects of differences in panicle branching patterns on grain filling, we evaluated the grain phenotype of Koshihikari and the NILs. Increasing panicle branching by introducing both *Pbl6*^ST-1^ and *Gn1a*^ST-1^ lowered thousand-grain weight (Fig. 5B). Comparing the thousand-grain weight between NIL-*Gn1a*^ST-1^ + *Pbl6*^ST-1^ and NIL-*Gn1a*^ST-1^ + *Prl5*^ST-1^ + *Pbl6*^ST-1^ suggested that the introduction of *Prl5*^ST-1^ raises grain weight (Fig. 5B). Since grain weight is often affected by the moisture content of seeds, we next conducted 3D analyses by X-ray CT of the panicle and calculated the corresponding seed volume. Compared to Koshihikari, grains had a smaller volume in NIL-*Gn1a*^ST-1^ + *Pbl6*^ST-1^ and NIL-*Gn1a*^ST-1^ + *Prl5*^ST-1^ + *Pbl6*^ST-1^, which produced more grains (Fig. 5C). Indeed, we observed more small-volume grains in these NILs compared to Koshihikari, suggesting that the photosynthetic capacity of Koshihikari may not be sufficient to fill all grains produced. The introduction of other alleles identified from QTLs that affect the photosynthetic rate, such as *NARROW LEAF1* (*NAL1*), could be an effective means to reduce the number of small-volume grains (Nakano *et al.* 2017, Takai *et al.* 2013). Notably, grain volume exhibited a trend toward larger values in NIL-*Gn1a*^ST-1^ + *Prl5*^ST-1^ + *Pbl6*^ST-1^ compared to NIL-*Gn1a*^ST-1^ + *Pbl6*^ST-1^, although the grain number was similar (Figs. 2H, 5C). This result suggests that *Prl5*^ST-1^ improves grain filling.

## Discussion

**Fig. 6.**
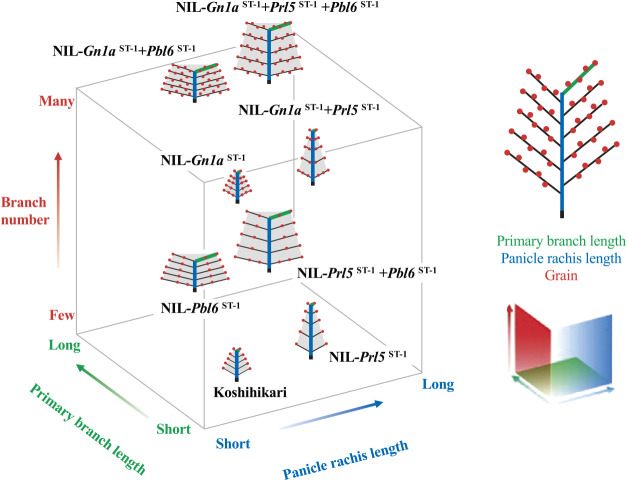
Three-dimensional landscape of panicle architecture determined by the alleles harbored at *Prl5*, *Pbl6*, and *Gn1a*.

In this study, we aimed to determine how the number of panicle branches and panicle length are coordinately regulated and discovered that these two traits were independently regulated by *Prl5*, *Pbl6*, and *Gn1a* without any trade-off. We demonstrated that various combinations of the developmental regulatory genes *Prl5*, *Pbl6*, and *Gn1a* produce diverse panicle branching patterns (Fig. 6). This result suggests that *Prl5*, *Pbl6*, and *Gn1a* are key genes that play an important role in the diversification of rice panicle morphology.

One reason to explain how the combination of the three alleles regulate panicle length and panicle branching number without any obvious trade-offs might lie in the different expression patterns of each gene (Agata *et al.* 2020, Li *et al.* 2013). Spatial and temporal patterns of gene expression are strictly controlled during panicle development (Supplemental Fig. 2), which may allow the independent control of each organ development. *Pbl6* was expressed during the earlier stages of panicle development (Supplemental Fig. 2B, 2E) and has a strong effect on the entire inflorescence meristem and pleiotropically regulates various organs (Agata *et al.* 2020, Ikeda-Kawakatsu *et al.* 2009, Ookawa *et al.* 2010). By contrast, *Prl5* was expressed at later stages (Supplemental Fig. 2B, 2D) and exhibits no pleiotropic effects and specifically regulates panicle rachis length. The different expression patterns of *Prl5* and *Pbl6* contribute to the different actions of *Prl5* and *Pbl6*. *Prl5* increases only panicle length, but *Pbl6* increases panicle length and branch number. *Gn1a* is expressed from earlier stages to later stages (Supplemental Fig. 2B). Cytokinins positively regulate cell division, while gibberellins positively regulate cell elongation, which is usually associated with cell differentiation (Veit 2009). In addition, Knotted1-like homeobox (KNOX) proteins regulate the balance between cytokinin and gibberellin activity in the meristem (Jasinski *et al.* 2005). A high cytokinin/low gibberellin ratio is thought to be important for preventing cell differentiation and thus maintaining stem cell fate. The interplay between KNOX transcription factors and the cytokinin/gibberellin ratio is at the core of organogenic competence (Shani *et al.* 2006, Veit 2009). Prl5 is involved in gibberellin biosynthesis, while Gn1a involved in the degradation of active cytokinins (Agata *et al.* 2020, Ashikari *et al.* 2005). In the primary branch initiation stage shown in the second image from the left of each panel in Supplemental Fig. 2C and 2D, the expression domains for *Gn1a* and *Prl5* are similar. In the region where primary branches differentiate, *Gn1a* and *Prl5* are expressed. This observation suggests that a low cytokinin/high gibberellin ratio is maintained in the differentiated region of primary branches. Thus, the mechanism that balances cytokinin and gibberellin levels, which has been demonstrated in shoots and during root formation (Jasinski *et al.* 2005), was also proposed to exist in panicle morphogenesis. The fine-tuning of the expression pattern of key developmental regulatory genes appears to independently control the development of each organ that comprises the panicle and produce diverse panicle architectures of natural rice variation.

Care must be taken during QTL pyramiding not to combine genes involved in similar mechanisms to avoid the negative effects that might arise from stronger alleles (Yano *et al.* 2015). The functions of the proteins encoded by *Prl5*, *Pbl6*, and *Gn1a* are different, which may also contribute to the lack of trade-off relationship between panicle length and branch number. Furthermore, the improvement of panicle architecture by combining the alleles of the three genes used in this study presented no trade-off relationship with other yield-related traits, except for panicle number. Although panicle number decreased slightly, the total yield increased due to improved panicle architecture. Therefore, we propose that combinations of *Prl5*^ST-1^, *Pbl6*^ST-1^, and *Gn1a*^ST-1^ contribute to yield improvement.

This study is the first to determine the detailed effect of different panicle branching patterns on grain filling using materials with a uniform genetic background and different panicle phenotypes. Detailed observations of panicle branching patterns and their influence on grain filling revealed the genetic factors related to both panicle architecture and grain filling. We unexpectedly discovered that *Prl5* affect grain filling. Indeed, the thousand-grain weight tended to increase upon the introduction of *Prl5* (Fig. 5B). CT scanning of panicles revealed that, despite being in the same genetic background, having the same grain shape, and producing almost the same grain number, differences in panicle branching patterns resulted in greater seed volume in NIL-*Gn1a*^ST-1^ + *Prl5*^ST-1^ + *Pbl6*^ST-1^ compared to NIL-*Pbl6*^ST-1^ + *Gn1a*^ST-1^ (Fig. 5C). Since the panicle length continues to elongate until the latter stage of the ripening period due to the introduction of *Prl5*^ST-1^, we hypothesize that continuous grain filling over a long period is possible. Because *Prl5* is expressed in the vascular bundles of the panicle rachis (Agata *et al.* 2020), the high accumulation of gibberellins caused by *Prl5*^ST-1^ may affect cell elongation and proliferation. As a result, the vascular bundles of the panicle rachis may become thicker and improve grain filling. Another possibility is that the elongation of the primary branches at the base of the panicle increases the distance and spread between branches, possibly improving grain filling at the base of the panicle. We conclude that *Prl5* might improve grain filling, although it does not affect panicle branching number. Further research will identify novel genetic factors contributing to grain filling without negative effects on panicle branching patterns. Exploring the relationship between the number and thickness of branches will be interesting. We also need to clarify the ideal panicle branching pattern that leads to improved grain filling by comparing the grain volumes between grains attached at the base and the tip of the panicle using our NILs.

Elucidating the genetic mechanisms underlying rice panicle development reveals how diverse plant morphologies are generated. This work also contributes to breeding by showing how developmental regulatory genes can be used to design an ideal plant architecture to enhance productivity.

## Author Contribution Statement

A.A. and T.H. designed the research; A.A., M.A., Y.S., H.K., and T.H. performed the experiments; A.A. and T.H. analyzed the data; A.A. and T.H. wrote the paper.

## Supplementary Material

Supplemental Figures

Supplemental Table
