# Supplementary material for: Designing rice panicle architecture via developmental regulatory genes
Source: Breed Sci. 2023 Mar 21;73(1):86–94. doi: 10.1270/jsbbs.22075 (PMC10165343; doi:10.1270/jsbbs.22075)
Supplement: Supplementary file 1 — Supplemental Figures [file 73_086_s1.pdf]

doi: 10.1270/jsbbs.22075

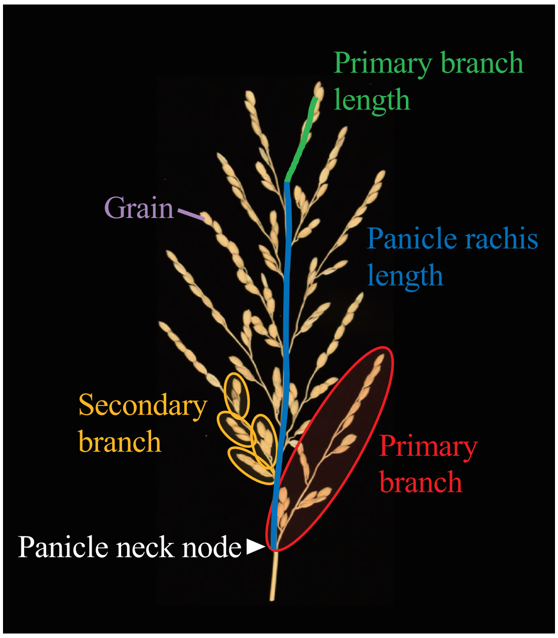

Supplemental Fig. 1. Rice panicle structure

A typical rice panicle. Panicle length is the sum of the panicle rachis length (blue) and the tip primary branch length (green). The primary branch (red) develops from the panicle rachis (blue), and each primary branch gives rise to secondary branches (yellow). Grains (purple) are attached at the tip of each branch.

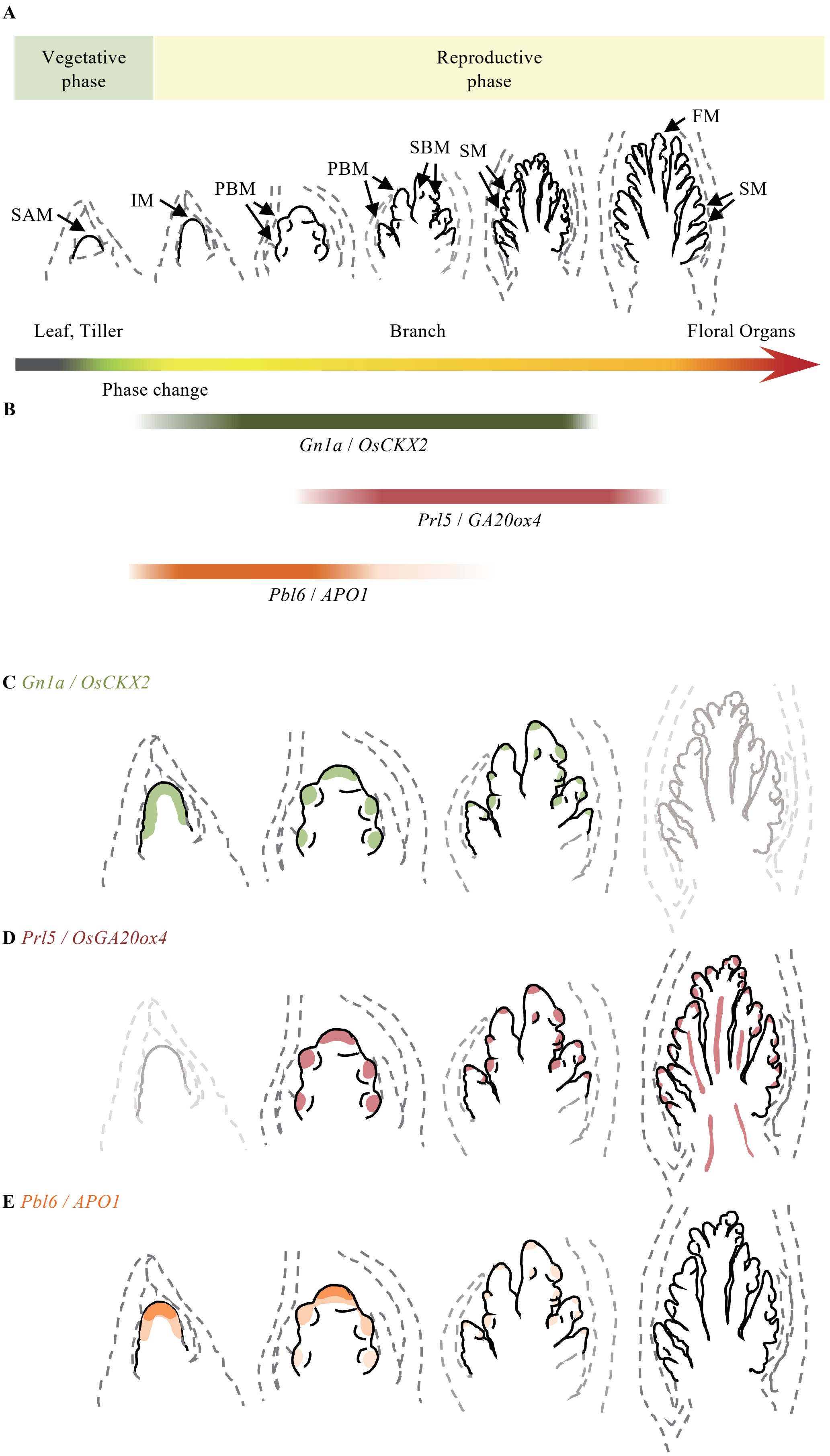

A
Vegetative phase
Reproductive phase
SAM
IM
PBM
PBM
SBM
SM
FM
SM
Leaf, Tiller
Branch
Floral Organs
Phase change
B
Gn1a / OsCKX2
Prl5 / GA20ox4
Pbl6 / APO1
C Gn1a / OsCKX2
D Prl5 / OsGA20ox4
E Pbl6 / APO1

Supplemental Fig. 2. Expression patterns of *Gn1a*, *Prl5*, and *Pbl6*

(A) Schematic diagram depicting the key developmental stages of rice panicle morphogenesis. SAM, shoot apical meristem; IM, inflorescence meristem; PBM, primary branch meristem; SBM, secondary branch meristem; SM, spikelet meristem; FM, floral meristem. **(B)** Expression timing of *Gn1a*, *Prl5*, and *Pbl6*. The length of each color bar roughly defines the temporal range of expression. This figure is based on previous studies (Agata *et al*. 2020, Ikeda-Kawakatsu *et al*. 2009, Li *et al*. 2013). **(C-E)** Spatial expression patterns of *Gn1a* (**C**), *Prl5* (**D**), and *Pbl6* (**E**) at the early stage of panicle development. Each expression area is colored. This figure is based on previous studies (Agata *et al*. 2020, Ikeda-Kawakatsu *et al*. 2009, Li *et al*. 2013). Stages with no data are filled in white.
