# Supplementary material for: Designing rice panicle architecture via developmental regulatory genes
Source: Breed Sci. 2023 Mar 21;73(1):86–94. doi: 10.1270/jsbbs.22075 (PMC10165343; doi:10.1270/jsbbs.22075)
Supplement: Supplementary file 2 — Supplemental Table [file 73_086_s2.pdf]

doi: 10.1270/jsbbs.22075

Supplemental Table 1. Primer sequences used for genotyping NILs

Name	Gene name	Forward Primer (5′ to 3′)	Reverse Primer (5′ to 3′)
qGn1	*Gn1a*	CCTTGTCCCTTCTACAATGG	AGTTGAGCATGAGGAGCACT
RM18711	*Prl5*	ACCTGCTCACCACAATTTGATTCC	TTCGAGCAATCTAGCCTGAGAGAGG
RM18717	*Prl5*	CAGCCTTGGTAGCTGGATAATCG	CGTCTACTGCTCGAGAGAACTGTGG
qPbl6_4	*Pbl6*	CCAAGTACTCCCTCCGTTTC	CAATGTCCGGTGTCTCTAGC
qPbl6_2	*Pbl6*	GTAGAGTAGCAGTTGTTGACAT	GAAATGATGAACACTGTCCAAC
